# From Medical Analgesic to Recreational Use: A Rare Case of Acute Myelitis Caused by Laughing Gas Abuse

**DOI:** 10.7759/cureus.41018

**Published:** 2023-06-27

**Authors:** Aicha H. Moussa, Walid Mohamed, Saad Zidouh

**Affiliations:** 1 Emergency Department, Faculty of Medicine and Pharmacy, Military Hospital Mohamed V, Rabat, MAR

**Keywords:** vitamin b12 deficiency, myelitis, neurological manifestation, misuse, nitrous oxide

## Abstract

Nitrous oxide, commonly known as laughing gas, which is originally intended to be used in various industries as well as for legitimate medical purposes, has unfortunately come to be used recreationally. However, it is crucial to recognize that the seemingly innocent recreational use of nitrous oxide can have serious repercussions on one's health. When consumed in excessive quantities and for prolonged periods, this substance can lead to serious adverse effects. In this article, we shed light on the abuse potential of the substance by discussing a distressing case of a young nitrous oxide user who developed severe myelitis.

## Introduction

In the past few years, there has been a significant upsurge in the non-medical utilization of nitrous oxide, which has raised immense concerns in the field of public health. Recent epidemiological investigations have shed light on the alarming rise in recreational nitrous oxide use and its associated health implications. The prevalence of nitrous oxide misuse, particularly among young individuals, has witnessed a substantial surge, as indicated by several studies [1-2]. Nitrous oxide is used recreationally primarily for its ability to induce anxiolysis, accompanied by euphoria or, in some cases, a dissociative effect. The common method of inhalation involves the use of cartridges sold as food industry propellants. This emerging trend has become a matter of grave concern for healthcare professionals and public health authorities due to the significant risks posed by the recreational use of nitrous oxide to both individual health and public well-being.

Additionally, recent research has elucidated the specific adverse effects of nitrous oxide on the neurological system. Inhalation of nitrous oxide, particularly in high doses or for prolonged periods, has been linked to various neurological complications, including myelitis. Myelitis is a rare yet severe condition characterized by inflammation of the spinal cord, resulting in symptoms such as muscle weakness, sensory disturbances, and loss of motor function. This case report highlights how the misuse of nitrous oxide can lead to detrimental neurological consequences.

## Case presentation

**Figure 1:**
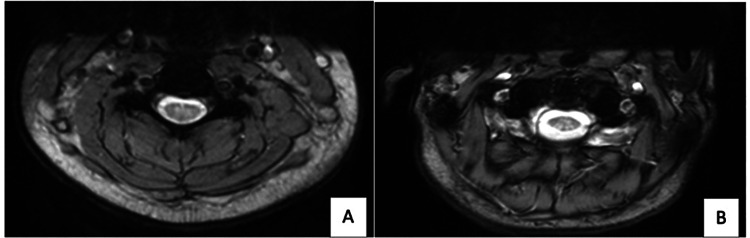
Spinal cord MRI of the patient with N2O (A) Axial T2 MRI showed symmetric and inverted V-shaped hyperintense signals in the dorsal columns of the cervical spinal cord. (B) Axial T2 MRI showed symmetric and inverted V-shaped hyperintense signals in the dorsal columns of the thoracic spinal cord MRI: magnetic resonance imaging

A 20-year-old female presented to the emergency department with gait problems. Apart from regular consumption of nitrous oxide from whipped cream siphon cartridges (approximately three bottles per day), she had no significant medical history. Over the past five days, she had been experiencing numbness-like paresthesias and muscle weakness in both lower limbs, with a progressive onset. The physical examination revealed a pyramidal syndrome characterized by areflexic flaccid paraparesis, along with a posterior cord syndrome manifesting as Lhermitte's sign and impaired proprioceptive sensitivity. Blood tests revealed normochromic macrocytic anemia and no signs of inflammation (white blood cells and C-reactive protein levels were within normal limits). A lumbar puncture was performed, but no abnormalities were detected. MRI showed extensive areas of T2 hypersignal affecting the posterior cords of the spinal cord, appearing as an inverted-V shape on extended cervical axial sequences from C1 to C2, C3 to C6, and D4 to D6 without the use of contrast (Figure 1).

This appearance indicated the presence of extensive myelitis with selective involvement of the posterior cords. Additionally, the patient exhibited vitamin B12 deficiency and hyperhomocysteinemia. The patient was transferred afterward to the neurology ward, where vitamin therapy and rehabilitation were initiated, and clinical improvement was subsequently observed.

## Discussion

Nitrous oxide, a gas commonly used as an anesthetic and analgesic in the medical field, as well as for culinary purposes in whipped cream siphons, is known for its oxidizing properties. The intense and prolonged consumption of nitrous oxide by young individuals for recreational purposes has been observed to be a high-risk behavior.

The subacute combined degeneration of the spinal cord observed in our patient is often recognized as a neurological manifestation resulting from the abuse of nitrous oxide [3]. The typical initial symptom is a tingling sensation in the distal parts of the lower and/or upper extremities. If this symptom is disregarded by the patient or not identified during the initial evaluation, individuals may subsequently develop gait ataxia (worsened in low lighting), frequent falls, or loss of ability to walk unaided. It is important to note that there may also be various clinical features associated with nitrous oxide misuse, including myeloneuropathy, isolated peripheral neuropathy, and even Guillain-Barré syndrome [4-7].

MRI scans of the cervical and thoracic spinal cord can serve the dual purpose of confirming the diagnosis and ruling out other possible causes. In most of the reported cases, MRI revealed an extensive hypersignal in the posterior cervical spinal cord on sagittal T2 STIR sections, along with an inverted V-shaped hypersignal on axial sections [8-9]. However, it is important to note that the condition cannot be distinguished by imaging alone, as many other conditions share exactly the same findings. The diagnosis is strengthened when the patient has a very clear history of recent, intensive nitrous oxide use.

As per the existing literature, the neurotoxic effects of nitrous oxide are linked to the oxidation of the cobalt ion found in vitamin B12, resulting in its inactivation. This process leads to a deficiency of vitamin B12 and hyperhomocysteinemia. Insufficient levels of vitamin B12 prevent the conversion of homocysteine into methionine, which is necessary for the methylation of myelin proteins. Disruption in this methylation process can result in demyelination in both the central and peripheral nervous systems. It should be emphasized that functional deficiencies can occur even when vitamin B12 levels are within the normal range. Therefore, evaluating homocysteine and methylmalonic acid levels can provide more precise and valuable information for confirming the neurotoxic effects of nitrous oxide [10].

Moreover, research has demonstrated that early administration of homocysteine and methylmalonic acid can contribute to the regression of symptoms in cases of nitrous oxide-induced neurotoxicity. However, the extent of recovery is dependent on the level of neuropathological damage to the spinal cord.

## Conclusions

Nitrous oxide abuse can lead to addictive behavior, especially as young individuals tend to underestimate its risks. Prolonged and intense consumption of nitrous oxide can result in severe neurological complications. Hence, it is essential to ensure prompt and individualized diagnosis and treatment for affected patients.
